# Diagnosis and treatment of fetal and pediatric age patients (0–12 years) with Wolff–Parkinson–White syndrome and atrioventricular accessory pathways

**DOI:** 10.2459/JCM.0000000000001484

**Published:** 2023-05-29

**Authors:** Loira Leoni, Gabriele Bronzetti, Diego Colonna, Giulio Porcedda, Alessandro Rimini, Massimo Stefano Silvetti

**Affiliations:** a Cardiology, Department of Cardio-Thoracic-Vascular Sciences and Public Health, University Hospital of Padua, European Reference Network for Rare and Low Prevalence Complex Disease of the Heart (ERN GUARD-Heart), Padua; b Cardio-Thoraco-Vascular Department, Sant’Orsola Hospital, University Hospital of Bologna IRCCS, Bologna; c Adult Congenital Heart Disease Unit, Monaldi Hospital, Naples; d Unit of Pediatric Cardiology, Anna Meyer Children's Hospital, Florence; e Giannina Gaslini Hospital IRCCS, Genoa; f Pediatric Cardiology and Cardiac Arrhythmia/Syncope Unit, and Bambino Gesù Children's Hospital, IRCCS, European Reference Network for Rare and Low Prevalence Complex Disease of the Heart (ERN GUARD-Heart). Rome, Italy

**Keywords:** atrioventricular accessory pathways, child, pediatric age, supraventricular tachycardias, Wolff–Parkinson–White syndrome

## Abstract

Overt or concealed accessory pathways are the anatomic substrates of ventricular preexcitation (VP), Wolff–Parkinson–White syndrome (WPW) and paroxysmal supraventricular tachycardia (PSVT). These arrhythmias are commonly observed in pediatric age. PSVT may occur at any age, from fetus to adulthood, and its symptoms range from none to syncope or heart failure. VP too can range from no symptoms to sudden cardiac death. Therefore, these arrhythmias frequently need risk stratification, electrophysiologic study, drug or ablation treatment. In this review of the literature, recommendations are given for diagnosis and treatment of fetal and pediatric age (≤12 years) WPW, VP, PSVT, and criteria for sport participation.

## Introduction

Atrioventricular (AV) accessory pathways (APs are frequently the cause of supraventricular tachycardias (SVT) in pediatric and fetal age. Therefore, this pathology is of great interest to all who care for children during the early stages of life. We present a review of the literature with current indications and recommendations for diagnosis, treatment, and sports eligibility in children.

## Anatomy and embryology

Ventricular preexcitation (VP) has a prevalence of 1–3/1000; in first-degree relatives of affected individuals, the estimated incidence is 5.5/1000.^1^ Wolff–Parkinson–White syndrome (WPW) occurs when the electrocardiographic sign – short PR and delta wave – corresponds to symptoms referable to PSVT: this occurs in about 50% of VP cases. Most VP carriers do not have structural heart disease; however, VP may be associated with congenital heart diseases (CHD) involving the AV junction such as Ebstein's anomaly, tricuspid atresia, and congenitally corrected transposition of the great arteries. The AP is the result of genetic or epigenetic errors affecting the AV myocardium.^2,3^ Proteins and receptors have been identified, such as Bmp2, Alk3, TBX2, and periostin, whose dysfunction leads not only to discontinuity of the annulus, but to this gap being occupied by fast-conducting tissue (Kent's bundle). Indeed, annulus discontinuity alone at the level of the tricuspid is demonstrable even in healthy patients, and it is not sufficient to explain all preexcitation syndromes. Malformations of the annulus may result in functional pathways with nodal-like properties, such as Mahaim fibers, but do not explain rapid pathways such as Kent. Furthermore, while the right APs penetrate through the annulus fibrosus, the left pathways more often ‘bypass’ the annulus with an epicardial course.

The formation of the fibrous annulus is not limited to the prenatal stages, but also affects those immediately following; this ontogenesis may explain the spontaneous resolution in >70% of neonatal tachycardias. Families with isolated or inherited WPW associated with cardiomyopathies have been described, with a higher incidence of multiple APs and increased risk of sudden cardiac death (SCD). The likelihood of multiple pathways increases with the complexity of the structural heart disease. A syndrome with myocardial hypertrophy, VP, supraventricular arrhythmias and conduction system defects has been described.^4,5^ The gene responsible is PRKAG217–19.^6^ VP is frequent in storage diseases such as Pompe^7^ and Danon disease (gene mutated: LAMP222), in mitochondrial diseases, and tuberous sclerosis with cardiac rhabdomyomas.^8^

Apart from the classic VP for AV APs, AV/fascicular connections (Mahaim), and node-fascicular/ventricular pathways are recognized, the discussion of which is beyond the scope of this paper.

## Atrioventricular reentry tachycardia in the fetal age

The incidence of arrhythmias in the fetal age is around 2%.^9^ Among the arrhythmias occurring during intrauterine life, excluding extrasystole, SVT are the most frequent (about 70% of cases). Untreated SVT are among the most important causes of heart failure with fetal hydrops and intrauterine death. In these cases, the prognosis is related to the gestational age of onset of the tachycardia, the mean ventricular rate of the tachycardia, the characteristics of the tachycardia (incessant or paroxysmal) and the presence of CHD (1–5% of cases).^9,10^

A fetal tachyarrhythmia is defined by a heart rate greater than 180 beats per minute (bpm) for >10 s. AV reentry tachycardia (AVRT) through an AP is diagnosed in presence of an AV ratio of 1:1 with ventriculo-atrial (VA) interval < AV interval.^11^

AVRT is by far the most frequent SVT in the fetal period (60–90% of cases). The heart rate is generally between 220 and 300 bpm. In the majority of cases, AVRT in the fetal period is orthodromic.^12,13^

The age of onset may vary between 21 and 34 weeks of gestation (most often between 24 and 32).^10^ Embryologically, the cardiac conduction system is functionally developed around the 16th week of gestation. At this stage of fetal life, an AP may form. Usually, in the course of fetal growth, the presence of an AP is transient and the abnormal muscle fascicle will spontaneously disappear. If this does not occur, the persistence of the AP may lead to PSVT.

Early recognition of the presence of a tachyarrhythmia is of paramount importance to initiate appropriate pharmacological treatment, achieve restoration of sinus rhythm and avoid the development of fetal heart failure. Heart failure and fetal hydrops worsen the prognosis and increase mortality, making even antiarrhythmic therapy in some cases ineffective.^14^

The diagnosis of AVRT in the fetus is made by fetal echocardiography. Despite the development of new innovative techniques, such as magnetocardiography, echocardiography is still the main tool not only for diagnosing the presence of a fetal arrhythmia, but also for identifying its type, assessing its main features and hemodynamic consequences. It is also possible to rule out the presence of a concomitant CHD or intracardiac tumors. The most used echocardiographic techniques are M-mode and Doppler assessment.^15^

## Diagnostic assessment

VP is often asymptomatic in children. In these patients, noninvasive assessment is performed approximately once a year, including cardiological examination and electrocardiogram, and, periodically, Holter, exercise test (>6 years) and echocardiography.

In the ECG, the characteristics of the delta and R waves during sinus rhythm and that of the P wave during tachycardia allow the localization of the AP with good approximation. Reliable algorithms exist.^16,17^

The echocardiogram, once associated structural or CHD have been ruled out, must verify, during follow-up, the occurrence of dysfunction from contractile dyssynchrony.

The finding of sudden and complete loss of preexcitation during exercise test or Holter, at physiological heart rates, is a positive predictive parameter.^18^ This loss is due to high refractoriness of the AP (i.e. poor conduction capacity of the pathway). However, intermittent VP, although less frequently associated with high-risk pathways, does not exclude the risk of SCD for which careful risk stratification is required.^19–21^

Arrhythmias were diagnosed in 10% of asymptomatic patients on the Holter/Event Recorder.^18^

In patients with WPW, and in those with AVRT due to concealed APs, undergoing drug treatment, noninvasive diagnostics are useful for monitoring its efficacy and safety.

However, noninvasive evaluation does not exclude potentially high-risk arrhythmias,^22^ so electrophysiological evaluation is required for risk stratification.

In untreated preexcitation/WPW patients the estimated risk of SCD is 0.9–2.4 per 1000 patient/year.^23^ The patients at higher risk of SCD are the symptomatic ones, with a risk of 0.0025 per patient/year, that is 3% over the lifetime.^24^ Progression from asymptomatic to symptomatic probably increases the risk of SCD. In children the risk is low, but probably higher than in adults, and the reported incidence is about 1.93 per 1000 patient/year.^25^ The overall incidence is 1.1 per 1000 patient/year in patients with normal heart, and 27 per 1000 patient/year in those with associated heart disease.^26^ The risk of sudden death is related to the anterograde conduction property of the AP and to untreated patients.^27,28^ However, it must be underlined that the true risk of sudden death in this population may be unknown, as many unexpected events occurring in normal hearts are not subjected to a careful WPW-oriented search. Furthermore, the risk of sudden death, especially in children, may be too low to allow an accurate estimate of positive or negative risk predictors.

SCD in patients with WPW is often related to physical activity due to the role of sympathetic stimulation in facilitating the AP conduction and inducibility of AVRT. However, it has also been reported at rest and during sleep in adolescent males.^21^ VP is responsible for approximately 1% of SCD in competitive athletes.^29^

In patients with palpitations or syncope, when the presence of AVRT is suspected, in addition to noninvasive diagnostics, an electrophysiological study (EPS) is required. The EPS may be transesophageal or intracavitary. The former can be performed as early as neonatal age, the latter upon reaching a weight of 10–15 kg. The intracavitary EPS can also assess the VA conduction through the AP and can accurately localize the AP. In symptomatic children under 8 years of age, the study of atrial vulnerability can be avoided.

In patients with asymptomatic VP, the EPS is useful for arrhythmic risk stratification.^1^ It should be performed around the age of 8–10 years, including assessment of atrial vulnerability at rest and during adrenergic stress (exercise or isoproterenol infusion). During the atrial vulnerability test, a burst of decreasing cycle pulses at a very high rate is delivered to induce atrial fibrillation (AF), in order to assess the conductivity of the AP, which lacks the decremental conduction of the AV node. AF can be induced in 25–30% of young people.^30^ Measuring the refractoriness of the AP is useful for risk stratification. A low refractoriness (high conduction velocity) of the overt AP, the so-called SPERRI (Shortest Pre-Excited R–R Interval), which can be measured during preexcited AF, if inducible, or during 1:1 conduction on the AP, in case of noninducible AF, is indicative of high risk of SCD. In these asymptomatic patients, ablation may be indicated (1) (see section 5). The presence of multiple APs is another risk factor.

The SPERRI would show high sensitivity (88–100%) and high negative predictive value, but low specificity (<75%).^31^ The AP refractory period (APERP) appears to be less predictive of life-threatening events, whereas it correlates better with AVRT inducibility. Di Mambro *et al.*^32^ found that symptomatic and asymptomatic pediatric patients have the same potential risk of SCD in the presence of inducible AF and that the AP location is not predictive of adverse events.

In asymptomatic children <8 years old with accessory pathways, risk stratification is not recommended.^23^ The EPS for risk stratification in asymptomatic patients can be repeated after puberty to assess possible changes of refractoriness and vulnerability.^33,34^ This may also be related to the dynamic nature of these parameters that are generally collected during one single procedure. Italian protocols for competitive sport participation (COCIS 2017) take into account electrophysiological parameters in addition to clinical data. SPERRI and/or APERP >250 ms at baseline and ≥210 ms during exertion/isoproterenol on transesophageal or endocavitary EPS allow competitive sport participation. In prepubertal children (<12 years) it is reasonable to adopt less restrictive criteria considering theoretically at risk an AF induced with SPERRI ≤ 210 ms at rest.^35^

## Drug therapy

### Drug therapy of fetal atrioventricular reentry tachycardia

The most widely used therapeutic technique to treat tachycardias in the fetus is the transplacental use of antiarrhythmic drugs. The use of antiarrhythmic drugs in pregnancy is always off-label but is now widely supported in the international literature; informed consent must be obtained from the pregnant woman. The clinical therapeutic program should be considered according to gestational age and shared with the other specialists.

**Fig. 1:**
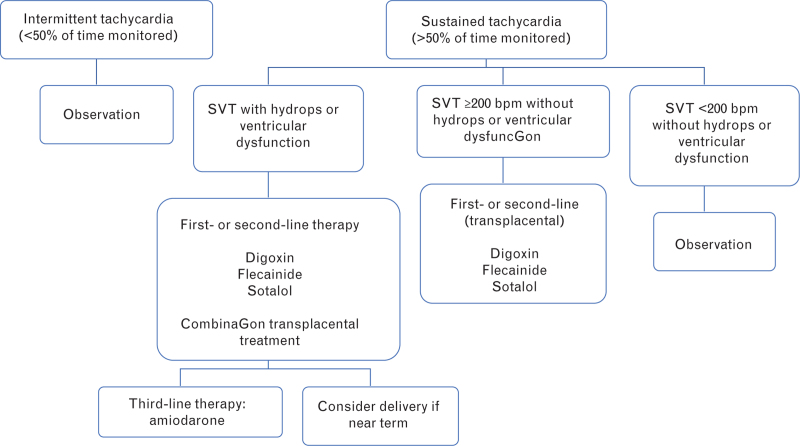
Maternal therapy for in-utero management of fetal tachycardias. SVT, supraventricular tachycardia.

Pregnant patients should undergo treatment in third-level or specialized centers with close clinical-instrumental monitoring (Fig. 1).

To allow transplacental passage of the antiarrhythmic drug and the achievement of adequate doses in the fetus, very high dosages must be given to the mother due to the high hepatic metabolism of the pregnant woman. The dosage must be increased gradually in order to avoid maternal and fetal side effects^36^ (Table 1).

**Table 1:** Drug treatment of fetal and childhood tachycardia

Drug	Fetal PSVT	
	Therapeutic maternal dose range	Therapeutic level
Digoxin	LD: 1200–1500 μg/24 h i.v., divided every 8 h MD: 375–750 μg/daily divided every 8–12 h p.o. (fetal intramuscular dose: 88 μg/kg q12 h, repeat 2 times)	0.7–2.0 ng/ml
Flecainide	100–300 mg/daily divided every 8–12 h p.o.	0.2–1.0 μg/ml
Sotalol	160–480 mg/daily divided every 8–12 h p.o.	Levels not monitored
Amiodarone	LD: 1800–2400 mg/daily divided every 6 h for 48 h p.o.; lower (800–1200 mg p.o.) if prior drug therapy MD: 200–600 mg/daily p.o. Consider discontinuation of drug and transition to another agent once rhythm is converted or hydrops has resolved	0.7–2.8 μg/ml
Propranolol	60–320 mg/day divided every 6 h p.o.	25–140 ng/ml
	**Children PSVT**	
**Drug**	**Dosage per body weight (children)**	**Features prompting lower dose or discontinuation**
Flecainide	LD 1–2 mg/kg in 10 min i.v. MD 2–6 mg/kg/daily divided every 8–12 h p.o.	QRS duration increase 0.25% above baseline
Propafenone	LD 0.5 mg/kg in 10 min i.v. MD 10–15 mg/kg/daily divided every 8 h p.o.	QRS duration increase 0.25% above baseline
Verapamil	LD 0.1–0,2 mg/kg in 5–10 min IV MD 2–8 mg/kg/daily divided every 8 h p.o.	Bradycardia
Sotalol	LD 0.2–1.5 mg/kg in 15 min i.v. MD 2–8 mg/kg/daily divided every 8–12 h p.o.	QT interval ≥500 ms
Amiodarone	LD: bolus 5 mg/kg in 30–60 min i.v., MD 5–10 mg/kg/daily i.v. MD 10–20 mg/kg/daily p.o. divided every 12 h for 10 day then 5–10 mg/kg/daily p.o.	QT interval ≥500 ms
Propranolol	LD 0.025–0.2 mg/kg in 10 min i.v. MD 1–6 mg/kg/daily divided every 6–8 h p.o.	Bradycardia
Metoprolol	MD 1–2 mg/kg/daily divided every 12 h p.o.	Bradycardia
Nadolol	MD 0.5–2 mg/kg/daily divided every 12–24 h p.o.	Bradycardia

i.v., intravenously; LD, loading dose; MD, maintenance dose; p.o., orally; PSVT, paroxysmal supraventricular tachycardia.

First- or second-line drugs are digitalis, flecainide and sotalol.^10,37,38^ For many centers, digitalis is the drug of first choice because of its safety profile, its long history of use during pregnancy and familiarity with its use. Flecainide is considered safe due to the absence of significant side effects in the fetus. This drug has proven to be extremely effective in the treatment of AVRT. Flecainide must be used with caution in cases of ventricular dysfunction, as it may worsen fetal heart failure or cause conduction disturbances and ventricular arrhythmias.^38^

In cases of AVRT refractory to treatment (after at least 7 days of administration of the drug chosen in the first instance at the maximum dosage), a combination of two drugs (flecainide/sotalol/digitalis) can also be used. When beta blockers are used as first- or second-line therapy, fetal growth must be monitored as it may be slowed.

Amiodarone has more adverse effects for mother and fetus than other drugs and should be reserved as third line for the treatment of nonresponsive tachyarrhythmias in the presence of heart failure with hydrops. Amiodarone therapy should be discontinued as soon as possible when the fetal heart rate decreases or the SVT is interrupted.^39,40^

Verapamil and procainamide cannot be used for the treatment of fetal tachyarrhythmias.

Once fetal heart rhythm control has been achieved, drug therapy should be continued until delivery. After birth, a transesophageal EPS can be performed, after drug wash-out of approximately 5 half-lives, to confirm the diagnosis and initiate antiarrhythmic therapy at dosages appropriate for the newborn.^41^

### Drug therapy of atrioventricular reentry tachycardia in children

#### Acute episodes

The treatment of acute episodes of AVRT consists of interrupting conduction through the AV node or AP.^42,43^

Therapeutic management changes according to clinical presentation: in heart failure or cardiogenic shock patients, synchronized external electrical cardioversion (0.5–2 J/kg) or adenosine are indicated. In centers with pediatric electrophysiology, transesophageal atrial pacing may be performed. The newborn with PSVT may be totally asymptomatic, but if the AVRT is of long duration, symptoms such as irritability, crying, and inappetence may occur, which are nonspecific and common to other neonatal conditions. The newborn is therefore particularly at risk of heart failure because neonatal AVRT may be misdiagnosed. Acidosis must be corrected.

When the patient is in good clinical condition and there are no signs of heart failure/cardiogenic shock, vagal maneuvers (such as the ‘diving reflex’) often interrupt the arrhythmia.^44^ The drug of first choice is adenosine, as a rapid bolus, followed by rapid infusion of saline. The therapy can be repeated and incremented (0.1–0.25 mg/kg). If adenosine is ineffective, long-acting antiarrhythmics are required. Intravenous (i.v.) Class IC drugs are first choice to be administered with close electrocardiographic monitoring. Amiodarone i.v. should be reserved for refractory cases or in the presence of heart failure or reduced systolic function. Nondihydropyridine calcium antagonists are contraindicated under 1 year of age because of the risk of electromechanical dissociation. Intravenous drugs are listed in Table 1.

## Prophylaxis of recurrences

**Fig. 2:**
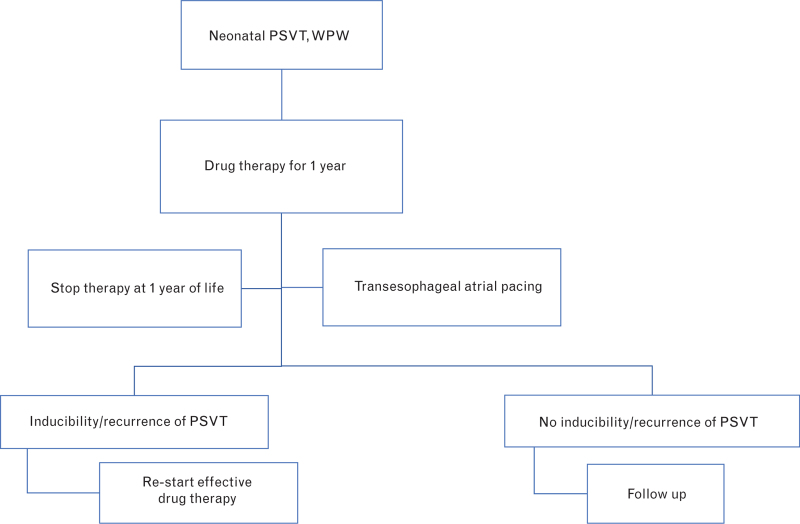
Therapeutic strategy in infants with PSVT. See text for further details. PSVT, paroxysmal supraventricular tachycardia; WPW, Wolff–Parkinson–White syndrome.

The transesophageal EPS can be performed to confirm the efficacy of antiarrhythmic therapy in the newborn/infant: noninducibility of AVRT during therapy predicts the absence of clinical recurrences during follow-up, whereas noninducibility after therapy discontinuation indicates the disappearance of the arrhythmogenic circuit.^45,46^ Chronic antiarrhythmic prophylaxis must be continued until at least the age of 1 year for WPW and PSVT. Further, in WPW and PSVT patients, antiarrhythmic prophylaxis should be carried out as long as AVRT recurs or remains inducible at transesophageal EPS (Fig. 2).

The spontaneous inducibility of AVRT decreases and the AP may disappear electrocardiographically and clinically during the very first years of life in about 50% of cases. Therefore, ablation should only be considered in selected cases (ineffective drugs, refractory heart failure),^46^ as the natural history of neonatal WPW and the high risk of ablation under 15 kg advises caution (see section 5).

Class IC antiarrhythmics are the drugs of first choice. Propafenone was effective in 70–90% of cases, and flecainide in 60–100%.^47^ The oral dosages are given in Table 1.

Class IC drugs and amiodarone can also be used in combination with beta blockers. A combination that has proved effective is flecainide and sotalol.

Flecainide is the most widely used drug with a good safety profile and good tolerability. Episodes of incessant SVT, more rarely ventricular tachycardia or severe bradycardia have been described for flecainide.^48^

For propafenone, the incidence of side effects, especially gastrointestinal^49^ is about 27%, while proarrhythmic effects are rare (<1%).

When arrhythmias are drug-resistant, sotalol (efficacy 63–100%, adverse effects 22%, proarrhythmia 9%) and amiodarone (efficacy about 90%, adverse effects up to 40%) have been used, also in combination with class I C antiarrhythmics, using lower dosages and with close ECG monitoring (QRS and QTc control). Chronic amiodarone, although better tolerated and with adverse effects more reversible than in adults, should be reserved for resistant cases or those with significant myocardial dysfunction. Among the side effects there are QTc prolongation, which may rarely cause torsades de pointes in children,^50^ thyroid and hepatic dysfunction, and photosensitivity. Not to be forgotten is the risk of hypoglycemia induced by beta blockers in the event of prolonged fasting or gastroenteritis.

## Transcatheter ablation

Transcatheter ablation of the AP has proved to be an effective and safe technique for treating PSVT/VP in pediatric patients. There are specific guidelines with indications based on age and body size.^51,52^

Procedural precautions are necessary to reduce the risks of complications, using suitable technologies that guarantee safety and efficacy, while reducing radiological exposure.^53^ Pediatric ablations must be performed under general anesthesia/deep sedation to minimize the child's emotional and psychological stress.

To ensure high standards of efficacy and safety, pediatric ablations should be performed by electrophysiologists experienced in pediatrics, in third-level centers with the most suitable technologies (radiofrequency, cryoablation, nonfluoroscopic mapping systems)^54^ and with the support of pediatric hemodynamists and cardiac surgeons for assistance in the event of serious complications.

Experimentally, it has been shown that the sizes of radiofrequency lesions on immature myocardium are acutely equal to those on adult myocardium, but, unlike in adults, in immature tissue they tend to grow over time with increased fibrosis.^55,56^ Therefore, we must carefully consider the appropriateness and timing of ablation in children, reserving procedures in infancy or early childhood only for patients with life-threatening arrhythmias and/or severe symptoms that cannot be controlled by drugs. Current guidelines rely primarily on the age^51^ and weight of the child^52^ to assess ablation risks. Ablations performed in children <4–5 years old and <15 kg are most at risk. The choice of energy to be used must also be guided by both the size of the patient and the type of substrate to be ablated. Indeed, radiofrequency may cause coronary lesions if the AP is close to a coronary artery,^56^ whereas cryoablation does not.^57^ In ablations close to the conduction tissue, cryoenergy is safer, with the same acute and chronic results, as it causes an initially reversible lesion during cryomapping.^58–61^ This allows us to evaluate the effect of freezing on both the normal conduction system and the arrhythmogenic substrate so that ablation can be performed safely.

To decide and perform an ablation in a child, one must take into account the natural history of the tachycardia, the risks of the arrhythmia and those of the procedure, and expected recurrences. In addition, one must consider the child psychology and the impact that chronic antiarrhythmic therapy or that of frequent recurrences may have on quality of life and performance compared to peers, leading to anxiety patterns and impairment of school performance in adolescence and youth.^62^ Therefore, the ablation choice must be multiparametric and balanced; it must not be made lightly, but neither must it be procrastinated over time while waiting for the child to become an adult in order to reduce the risks. Both choices could harm the child.

Although the low risk of having the arrhythmogenic substrate can be removed during the diagnostic session (i.e. the intracavitary EPS) in experienced hands, ablation should not be presented as a necessary completion of the interventional procedure. Further, in the child, the EP study is often transesophageal.

### Efficacy of ablation

In 2021, a multicenter registry reported 96% efficacy of transcatheter ablation of WPW.^63^ However, the efficacy varies depending on the location of the AP. The ablation efficacy of the left lateral APs is 98%, while that of the right APs and right-left septal pathways is between 88% and 90%.^64^

**Fig. 3:**
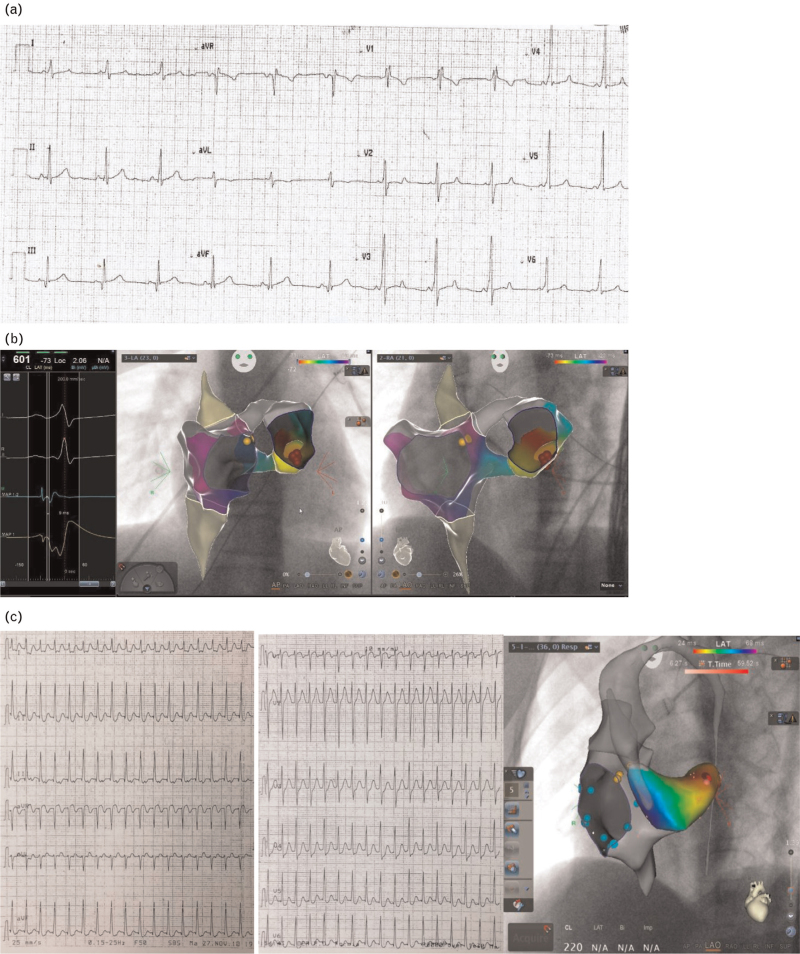
(a) ECG showing VP from a left posterolateral AP. (b) 3D-electroanatomic map of right and left atria (Carto Univu Biosense Webster Inc., diamond bar, CA). From left to right: ablation site local electrogram; left postero-lateral ablation site (red tags), on antero-posterior (middle panel) and left anterior oblique views (left panel). Orange tags: His bundle recording site. Trans-septal approach. (c) ECG showing PSVT (left panel) and 3D-electroanatomic map of right and left atria (Carto Univu Biosense Webster Inc., Diamond Bar, CA) (right panel). Left lateral concealed AP ablation site (red tags, left anterior oblique view). Retrograde transaortic approach. Orange tags: his bundle recording site; light-blue tags: tricuspid annulus. AP, accessory pathway; PSVT, paroxysmal supraventricular tachycardia.

**Fig. 4:**
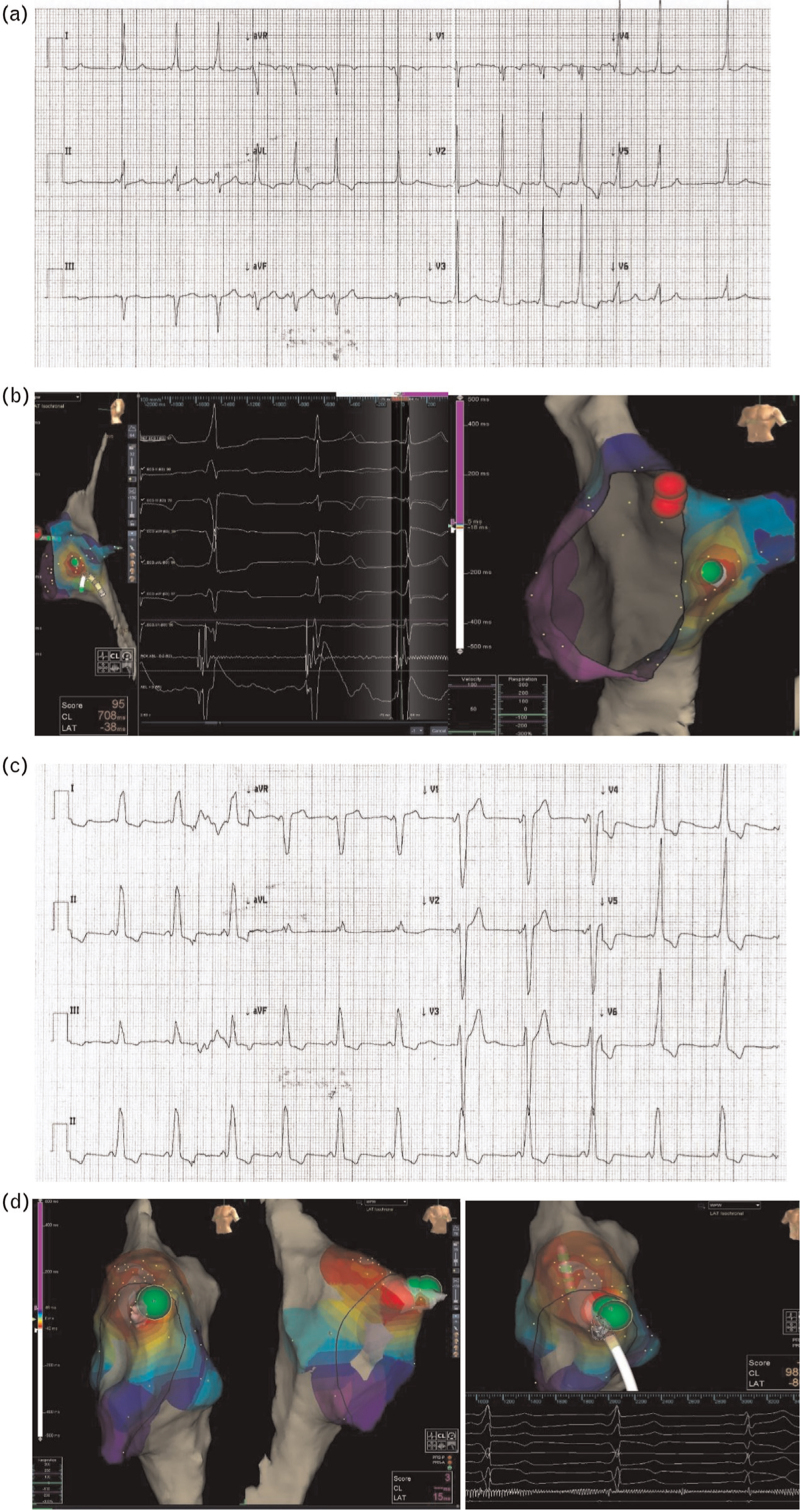
(a) ECG showing VP from a right mid-septal AP. (b) 3D-electroanatomic map of right atrium (EnSite Precision 5.0.1 Abbott Medical). Mid-septal cryoablation site (green tag). Red tags: His bundle recording site. From left to right, left lateral view, ECG and local electrogram at the ablation site showing the disappearance of the delta wave during cryomapping, left anterior oblique view. (c) ECG showing VP from a right antero-septal AP. (d) 3D-electroanatomic map of right atrium (EnSite Precision 5.0.1 Abbott Medical). From left to right, antero-septal cryoablation site (green tags), left and right anterior oblique views. Red tags: his bundle recording site. Last panel shows the disappearance of the delta wave during cryomapping. AP, accessory pathway; PSVT, paroxysmal supraventricular tachycardia; VP, ventricular preexcitation.

The ablation procedure of the APs is usually performed with radiofrequency (Fig. 3), reserving the ablation of the septal pathways at greater risk of damage to the conduction system for cryoenergy (Fig. 4). The use of nonfluoroscopic 3D mapping systems (Figs. 3 and 4)^65–67^ that can also combine magnetic resonance imaging or computed tomography images^68^ can improve efficacy and reduce complications as well as fluoroscopic exposure.^69–72^

Recurrences at 12 months differ according to the site of the APs with higher percentages for right lateral (25%) and septal (16%) pathways than left APs (5% for left lateral and 9% for left septal).^64^

Ablations of the left pathways can be performed with an anterograde approach through the patent foramen ovalis or with a trans-septal procedure, and retrogradely by a transaortic procedure.^65^ Data from the literature show no differences between the two approaches in terms of safety and efficacy,^53^ and the choice is generally left to the operator.^73^

### Safety and complications

Pediatric procedures in third-level centers with experienced staff and the use of state-of-the-art methods increased the effectiveness of the procedures and reduced the risk of complications. Adequate training is required.^74^ The risks related to ablation of an AP vary depending on the location of the pathway.

The risk of death related to radiofrequency ablation of an AP in pediatric patients is anecdotal: death was caused by cardiac perforation, coronary or cerebral thromboembolism, and ventricular arrhythmia. In the few cases reported in the literature, it seems to correlate with the presence of an underlying heart disease, with the patient's weight, and with an increased number of radiofrequency applications. The cumulative incidence was 0.22%, and that in children aged between 0.1 and 13.3 years was 0.12% in the presence of a normal heart and 0.89% in the presence of structural heart disease.^75,76^

The most common serious complications reported in the literature are AV blocks, cardiac perforation/pericardial perforation, coronary artery damage, thrombus formation and emboli. The risk increases inversely with patient weight and age and is greater in patients of lower weight.^64^

Radiofrequency risks of block are 0.5% for right bundle branch block, 0.1% for left bundle branch block, and about 1% for complete AV block.^1,77^ With the use of cryoenergy, the risk of AV block is practically none.^78^

The risk of coronary damage is 1–2% higher in procedures performed within the coronary sinus and cardiac veins.^68^ Studies in adult patients and animals suggest a relationship with higher energy levels (radiofrequency), multiple applications and the site of delivery (lateral and posterior atrioventricular groove).^56,79^ Cryoablation does not appear to cause coronary artery damage.^57^

The risk of cardiac perforation is approximately 0.6%,^64^ while the risk of thrombosis and embolism is 0.6–0.8%.^1,80^

Minor complications such as hematomas, pseudoaneurysms, arteriovenous fistulas and minor bleeding appear to be low and have an overall incidence of about 1–3%.^1,64,75,81^ In this context, the reduction in the number of catheters used during pediatric ablation thanks to new nonfluoroscopic mapping systems is of paramount importance.^65^

Intravenous antithrombotic prophylaxis with heparin is good practice during the left accessory ablation procedure. Antiplatelet therapy with acetylsalicylic acid may be combined and continued over the next 10–30 days.

Right-sided preexcitation, which manifests on ECG with a left bundle branch block appearance, may rarely cause ventricular dyssynchrony and left ventricular dysfunction, which resolves in most cases after ablation of the AP.^82–87^

### Indications

**Fig. 5:**
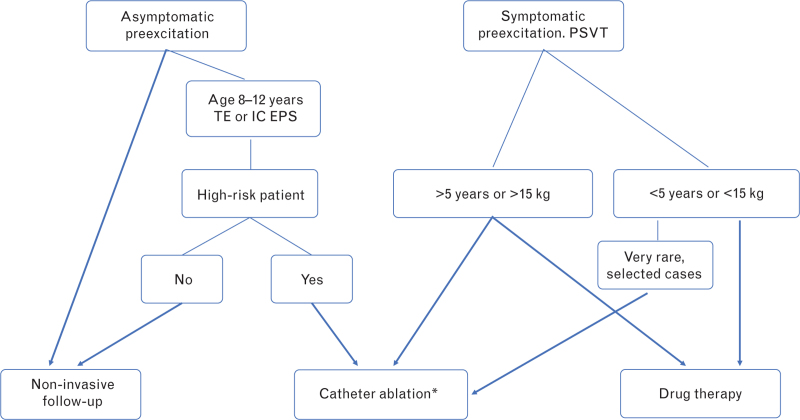
Flowchart of the management of children with preexcitation syndromes/PSVT after first year of life. See text and Table 2 for further details. A more detailed management algorithm of asymptomatic preexcitation patients can be found in reference 1. ^∗^ Careful assessment of the risk–benefit of ablation based on the patient's weight and size and the site of the accessory pathway. For high-risk asymptomatic patients, the ablation is always a Class II indication. EPS, electrophysiological study; IC, intracardiac; PSVT, paroxysmal supraventricular tachycardia; TE, transesophageal.

The indications for ablation procedures in children with WPW/VP/AVRT are given in Table 2. Figure 5 shows a flowchart of the management of children with preexcitation syndromes/PSVT after the first year of life.

**Table 2:** Indications for ablation procedures in pediatric patients with WPW/PSVT/VP^1,51,52^

Indications	Class of raccomandation, LOE
WPW and episode of aborted SCD	Class I, B/C
WPW and syncope combined with SPERRI during AF <250 ms or antegrade APERP during EPS <250 ms	Class I, B/C
WPW and recurrent and/or symptomatic PSVT and age >5 years	Class I, C
VP and palpitations with inducible sustained PSVT during EPS, age >5 years	Class I, C
PSVT, age >5 years, when chronic antiarrhythmic therapy has been effective in control of the arrhythmia	Class II A, C
PSVT, age <5 years (including infants), when antiarrhythmic therapy, including Classes I and III are not effective or associated with intolerable side effects	Class II A, C
Young (8–21 years) asymptomatic patient with persistent manifest preexcitation and SPERRI in atrial fibrillation ≤250 ms, taking into account the procedural risk factors based on the anatomical location of the pathway	Class II A, B/C
VP with ventricular dysfunction presumed due to dyssynchrony in larger (>15 kg) patients	Class II A, B
VP with syncope, without predictors (multiple APs, SPERRI during AF <250 ms or antegrade APERP during EPS <250 ms) of high risk for cardiac arrest in larger (>15 kg) patients	Class II A, C
Asymptomatic VP in larger (>15 kg) patients when the absence of VP is a prerequisite for participation in personal or professional activities	Class II A, E
Asymptomatic VP, age >5 years and >15 kg, no recognized tachycardia, and with predictors of low risk for cardiac arrest; risks and benefits of procedure and arrhythmia clearly explained	Class II B, C
Single or infrequent PSVT (no preexcitation), age >5 years	Class II B, C
WPW and recurrent and/or symptomatic PSVT and age <5 years	Class II B, C
Young (8–21 years) asymptomatic patient with persistent manifest preexcitation and inducible sustained PSVT, taking into account the risk/benefits of ablation	Class II B, C
VP caused by a fasciculoventricular accessory pathway	Class III, C
Asymptomatic VP, age <5 years	Class III, C
PSVT controlled with conventional antiarrhythmic drugs, age <5 years	Class III, C

AF, atrial fibrillation; APERP, accessory pathways effective refractory period; EPS, electrophysiological study; LOE, level of evidence; PSVT, paroxysmal supraventricular tachycardia; SCD, sudden cardiac death; SPERRI, Shortest Pre-Excited R–R Interval; VP, ventricular preexcitation; WPW, Wolff–Parkinson–White syndrome.

New systems (3D-mapping systems) and techniques (e.g. catheters), and safer energies (cryoablation, or newer sources), in third-level centers and experienced hands, may certainly allow an increase in ablation indications and procedures in the near future.

## Sport participation

Rhythm disorders represent a challenge for the pediatrician, the pediatric cardiologist and the sports physician in order to provide advice and appropriate indications for sport participation and regular exercise programs, considering the benefits associated with physical activity and the cardiovascular risk.

In Italy, the assessment of the health status of sport participants is the responsibility of the sports physician for competitive sport and of the pediatrician or sports physician for noncompetitive physical activity.^35,88^ Children between 0 and 6 years of age do not require medical certification for sport participation, except in specific cases indicated by the pediatrician.^88^

### Wolff–Parkinson–White syndrome/ventricular preexcitation

For noncompetitive sport participation in children <12 years of age, a distinction must be made between symptomatic or asymptomatic patients, with or without structural heart disease. In the presence of structural or CHD, refer to the specific heart disease guidelines.

Many children with VP are asymptomatic, but this may change over time.^1,89^ In asymptomatic children, without structural heart disease, the risk of developing AF or SCD is very low.^21,25,31,90^ In these patients, there are no contraindications for leisure-time physical activity and the EPS could be postponed until 8–12 years of age when AF becomes more frequent, and competitive sport participation begins.^35^ In case of asymptomatic APs with high refractoriness and low risk of SCD, sport participation is allowed even without ablation.

The symptomatic child without structural heart disease should undergo EPS and treatment to improve his quality of life and to eliminate the risk of SCD (which at all ages is higher in the symptomatic patient). Antiarrhythmic therapy is not a contraindication to leisure-time physical activity. Exercise should be discontinued if palpitations appear. Sports activities in which the potential loss of consciousness could be fatal should be avoided.

### Atrioventricular reentry tachycardia

Orthodromic AVRT, without VP on the surface ECG, is considered a ‘benign’ arrhythmia provided that structural heart diseases have been excluded. During physical activity, the heart rate of AVRT increases due to sympathetic stimulation and this may induce symptoms such as dizziness, fatigue or syncope even in the absence of structural heart disease. Therefore, physical activity should be interrupted as soon as palpitations occur.

It is important to rule out latent and intermittent preexcitation. Minimal or latent VP can be unmasked on ECG by simple interventions such as vagal maneuvers or intravenous adenosine to slow or block AV node conduction.

In AVRT without VP, noncompetitive sport participation is allowed even without treatment in patients who are asymptomatic or minimally symptomatic during tachycardia. All patients with AVRT should be instructed on how to safely perform vagal maneuvers to interrupt tachyarrhythmia. Exercise can be resumed after arrhythmia termination. Antiarrhythmic therapy is not a contraindication to leisure-time physical activity. If successful ablation of the arrhythmia is performed, physical activity can be resumed in most cases after 1 week (i.e. after healing of the vascular access sites).^91,92^

## Conclusions

WPW, PSVT, and VP are frequent pediatric arrhythmias that require proper diagnosis and treatment. WPW and VP carry a small but not negligible risk of sudden death, and noninvasive cardiac assessment and EPS are useful for risk stratification. Antiarrhythmic drug therapy is often effective in acute treatment and in chronic prophylaxis of PSVT. Transcatheter ablation is the treatment of choice for symptomatic and high-risk patients and is effective and safe in experienced centers.

### Conflicts of interest

There are no conflicts of interest.
